# Supplementary material for: Causal Relationship between Aging and Anorexia Nervosa: A White-Matter-Microstructure-Mediated Mendelian Randomization Analysis
Source: Biomedicines. 2024 Aug 16;12(8):1874. doi: 10.3390/biomedicines12081874 (PMC11351342; doi:10.3390/biomedicines12081874)
Supplement: Supplementary file 1 [file biomedicines-12-01874-s001.zip › Supplementary Figure/Figure S8/Leaveoneout plot of MR analysis for TL to ubm-b-1636.pdf]

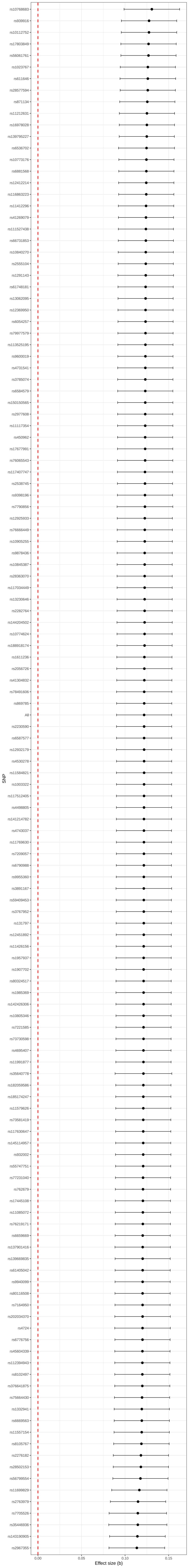
rs10768683
rs939916
rs10112752
rs17803849
rs56061761
rs1023767
rs611646
rs28577594
rs871134
rs11212631
rs16978028
rs139795227
rs6536702
rs10773176
rs6881568
rs12412214
rs116863223
rs11412296
rs41269079
rs111527438
rs66731853
rs10840270
rs2555104
rs1291143
rs61748181
rs13062095
rs12369950
rs6054257
rs79977579
rs113525195
rs9600019
rs4731541
rs3785074
rs6584579
rs150150565
rs2977608
rs11117354
rs450962
rs17677991
rs76065543
rs117407747
rs2538745
rs9398196
rs7790856
rs12925933
rs76666449
rs10905255
rs9878436
rs10845387
rs28363070
rs117034449
rs13230646
rs2282764
rs144204502
rs10774624
rs188918174
rs1611236
rs2056726
rs41304832
rs78491606
rs869785
All
rs2230590
rs6587577
rs12932179
rs4530278
rs11584821
rs1003322
rs117512405
rs4498805
rs141214782
rs4743037
rs11769630
rs7209057
rs6790988
rs9955360
rs3891167
rs59409453
rs3767952
rs131797
rs12451892
rs11426156
rs1957937
rs1907702
rs80324517
rs1985369
rs142426306
rs10805346
rs7221585
rs73730598
rs4695407
rs11991877
rs35640778
rs182059586
rs185174247
rs11579626
rs73581419
rs117630647
rs145114957
rs932002
rs55747751
rs77231040
rs762679
rs17445108
rs11085072
rs76219171
rs6659669
rs137901416
rs139669835
rs61405042
rs9940099
rs80116508
rs7164950
rs202034370
rs4724
rs6776756
rs45604339
rs112394943
rs8102497
rs376641875
rs75664430
rs1332941
rs6669563
rs11557154
rs8105767
rs2276182
rs28502153
rs56799554
rs11699829
rs2763979
rs7705526
rs35446936
rs143190905
rs2967355
SNP
0.00
0.05
0.10
0.15
Effect size (b)
